# Oxidative stress markers as a diagnostic tool in oral cancer and premalignant lesions: A systematic review

**DOI:** 10.34172/joddd.025.42130

**Published:** 2025-09-30

**Authors:** Sanaa Ahmed, Uzma Nasib, Muhammad Khalil Khan, Muhammad Ali, Obaid Ur Rahman, Nauyaan Ahmed Qureshi

**Affiliations:** ^1^Department of Oral Medicine, Jinnah Sindh Medical University, Karachi, Pakistan; ^2^Department of Biochemistry, Jinnah Sindh Medical University, Karachi, Pakistan; ^3^Department of Periodontology, Jinnah Sindh Medical University, Karachi, Pakistan; ^4^Department of Oral and Maxillofacial Surgery, Baqai Dental College, Baqai Medical University, Karachi, Pakistan; ^5^Department of Biochemistry, Swat Medical College, Swat, Pakistan; ^6^Intern, Agha Khan Hospital, Karachi, Pakistan

**Keywords:** Areca, Arecoline, Chromatin assembly and disassembly, Interleukin-6, Interleukin-8, Oral neoplasms, Reactive nitrogen species, Reactive oxygen species, Squamous cell carcinoma, Tumor suppressor protein

## Abstract

**Background.:**

Oral cancer is the eighth leading cause of cancer-related mortality worldwide, particularly prevalent in regions with high tobacco and betel nut consumption. The associated high mortality and morbidity rates could be reduced through early detection, prompting researchers to focus on identifying early markers of carcinogenesis.

**Methods.:**

This systematic review evaluated the effectiveness of oxidative stress markers as diagnostic tools. An electronic search was conducted in PubMed and Google Scholar to identify case‒control studies published between January 2000 and June 2024 that explored the use of oxidative stress markers as diagnostic biomarkers. A manual search was also performed in relevant journals, including oral oncology and oral diseases. Initially, 38 studies were screened, and after applying the inclusion criteria, only nine studies were included. The Newcastle-Ottawa Scale (NOS) tool was used to assess the risk of bias.

**Results.:**

Eight studies were conducted in India, while one was from Saudi Arabia. These studies analyzed oxidative stress markers in oral squamous cell carcinoma (OSCC), oral submucous fibrosis (OSMF), and leukoplakia. Control groups were matched based on age and sex, with only two studies also considering socioeconomic status. A significant difference (*P*<0.05) in oxidative stress marker levels was observed between cases and controls, particularly in patients with OSCC and OSMF.

**Conclusion.:**

Oxidative stress markers show promise as diagnostic and prognostic indicators. Standardized methodologies and therapeutic approaches targeting oxidative stress could enhance early detection and treatment, especially in resource-limited settings. However, the findings must be interpreted with caution due to methodological limitations, geographic bias, and the lack of inclusion of grey literature.

## Introduction

 The Global Cancer Statistics report from 2020 estimated 377,713 new cases of lip and oral cavity cancer diagnosed annually, resulting in 177,757 deaths worldwide that year. This cancer ranked as the eighth most common in both incidence and mortality among males.^1^ Oral squamous cell carcinoma (OSCC) is the second leading cause of cancer-related deaths in Southeast Asia, primarily due to betel nut and tobacco consumption.^2,3^ Other contributing factors, including genetic and environmental influences, also play a significant role in OSCC development.^3^ The documented precursors of OSCC include premalignant lesions such as erythroplakia and leukoplakia, with oral submucous fibrosis (OSMF) being the most prevalent condition among these patients.^4^

 OSMF is a common oral condition diagnosed in Southeast Asia, primarily due to the high consumption of betel nut in various forms. It is estimated that around 600 million people chew areca nut in the Asia-Pacific region.^3^ This habit of consuming betel quid with or without areca nut regularly stimulates an inflammatory response that consists of polymorphic infiltration and dilatation of the vessels due to the release of alkaloids, copper, flavonols, and tannins. Additionally, arecoline induces uncontrolled collagen synthesis, resulting in the formation of fibrous bands in the oral mucosa.^5^ These fibrous bands decrease the mouth opening and create an environment in which even minor trauma may lead to dysplastic changes.^6^

 The carcinogenesis pathways involve changes at various levels as a result of injury to the oral mucosa. These include genetic mutations resulting from changes in DNA methylation, histone covalent modifications, chromatin remodeling, and the effects of non-coding RNA on gene expression or the deactivation of inhibitory genes, such as p53, IL-1β, IL-6, and IL-8. The products of these processes may serve as proteomic markers and genomic markers.^7,8^ Then come the underlying metabolic changes as a result of coping with the injury, which are termed as metabolomic markers. These include the oxidative stress markers (reactive oxygen and reactive nitrogen species) that may arise after the oxidation of lipids. They have shown potential as precise and reliable markers of the neoplastic process.^7^ Under normal circumstances, ROS/RNS are neutralized into less reactive species, but due to impaired or compromised neutralizing mechanisms, these products may result in damage to the cell’s genetic material, i.e., DNA, membrane, and further cause the loss of function in tumor suppressor genes, leading to carcinogenesis and tumor progression.^9-11^ Their pivotal status in the formation and progression of neoplasia makes them the prime candidate for use as diagnostic and prognostic markers in susceptible individuals.

 The morbidity and mortality rates of oral cancer are high in countries with lower socioeconomic status and high consumption of betel nut.^3^ This has shifted physicians’ focus towards prevention and early diagnosis, which has started a new race to find markers that are cheap, valid, and reliable, indicating early changes within preventable limits in the dysplastic/paraneoplastic changes.^8-10^ Hence, this review includes both premalignant lesions (such as OSMF and leukoplakia) and malignant lesions (OSCC) to evaluate whether oxidative stress markers can detect changes across the oral carcinogenesis process. Since premalignant lesions precede oral cancer, assessing biomarkers in these conditions may provide early warning signals, enhancing their potential value as diagnostic and prognostic tools. The purpose of this systematic review was to assess the effectiveness of oxidative stress markers as a diagnostic test. The review was“What is the efficacy of oxidative markers as a diagnostic tool for early detection of oral cancer?”

## Methods

 This systematic review was registered with PROSPERO (CRD No: CRD42024565417) and adhered to PRISMA guidelines. The PICO framework (Population, Intervention, Comparison, Outcome) was used for study selection. Literature was searched in PubMed, Google Scholar, and PROSPERO for topic duplication and removed through the EndNote software. Additionally, manual searches were conducted in journals on oral diseases and oral oncology. The review period spanned from August 2024 to October 2024. Key words included “diagnostic markers,” “oxidative stress markers,” “oral cancer,” “OSCC,” “MDA,” “8-isoprostane,” and “premalignant lesions.” Ethical approval was obtained where required, and patient consent was ensured.

###  Inclusion criteria

1. Case‒control studies
2. Studies evaluating oxidative markers in histopathologically proven oral cancer patients compared to premalignant or healthy individuals
3. All articles published in English between 2000 and 2024

###  Exclusion criteria

1. Any studies conducted before 2000
2. Articles in languages other than English language

###  Data collection strategy

**Figure 1:**
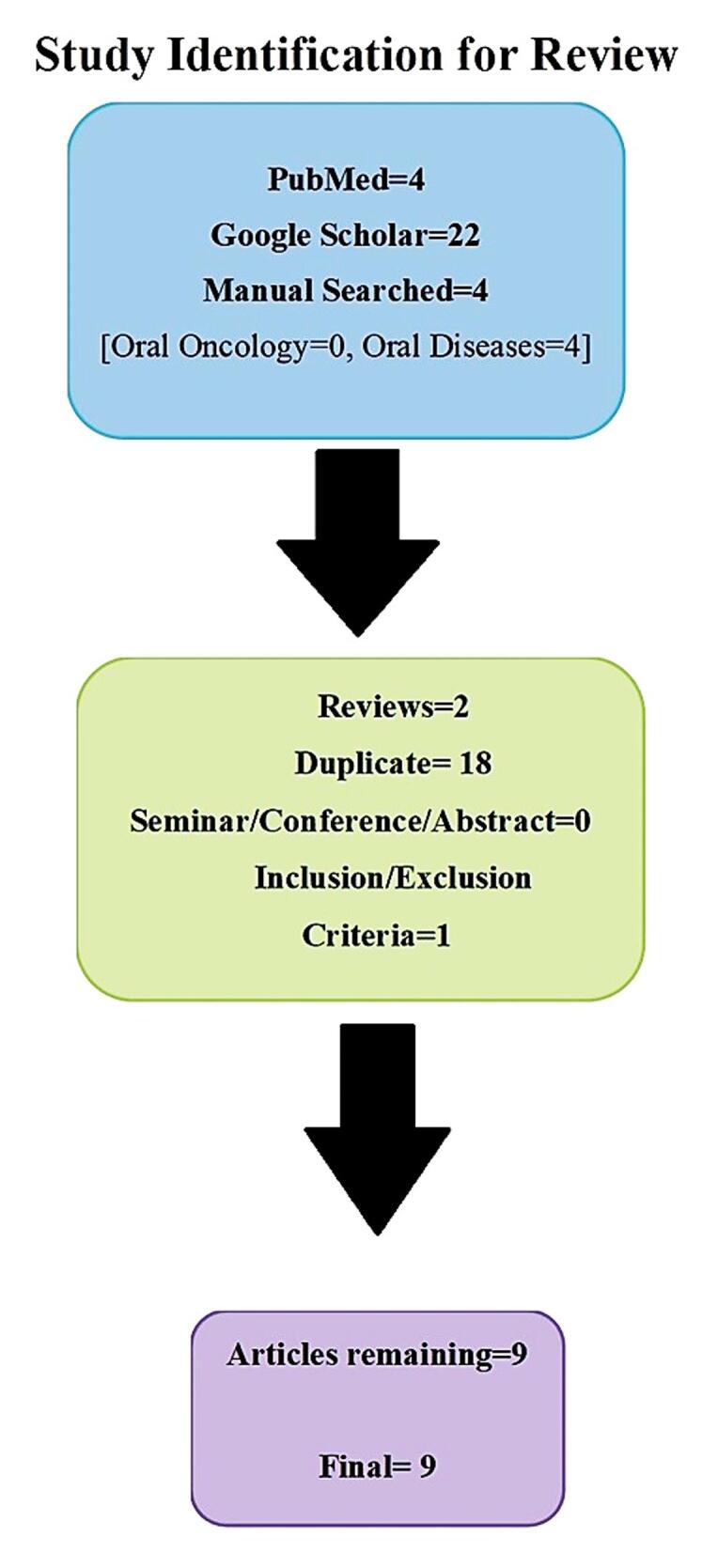
Flow chart showing selection steps for literature search

 Manual search and selection of studies for the review were completed according to the previously described criteria by the two authors (SA and UN). In phase 1, the reviewers reviewed the abstracts of the articles found through the search. Articles were further screened according to the inclusion criteria in phase two. In case of any disagreement, it was resolved through discussion. If the agreement could not be reached, then a third reviewer (MKK) made the final call to add or remove the study from the review (Figure 1). The final selection was based on consensus between all three authors. We did not calculate inter-rater reliability.

###  Comparator(s)/control

 Level of oxidative stress markers in healthy controls and patients diagnosed with premalignant lesions

###  Main outcome

 Comparing the level of oxidative stress markers in oral cancer vs. the level of markers in patients with premalignant lesions or healthy controls

###  Risk of bias (quality) assessment

 The Newcastle–Ottawa Scale (NOS) was used for case‒control studies. This tool evaluates three domains: the selection of cases and controls, the comparability of groups, and the ascertainment of exposure. Each study can score up to 9 stars; higher scores indicate lower risk of bias.

###  Strategy for data synthesis

 A comparative table will be generated based on the different oxidative stress markers explored in oral cancer and their significance at the 0.05 level.

## Results

 Nine case‒control studies were included, focusing on OSCC, OSMF, and oral leukoplakia (Table 1). These studies assessed markers such as 8-isoprostane, malondialdehyde (MDA), and sialic acid, primarily using serum and salivary samples. Matching criteria included age, sex, and socioeconomic status. Four studies also considered lifestyle factors such as smoking and betel quid chewing. The studies consistently reported elevated oxidative stress markers in oral cancer cases compared to controls, reinforcing their diagnostic relevance.

**Table 1:** Comparison of demographic and methodological aspects of the studies

**Author and year**	**Country**	**Objective**	**Number of groups**	**Case to control ratio**	**Group** **specification**	**Intervention**	**Duration of study**
Beevi et al/2003 ^12^	India	To evaluate the magnitude of oxidative stress and levels of nitric oxide in patients with oral cavity cancer by analyzing the levels of lipid peroxidation products, antioxidants, and nitric oxide products.	2	1:1 Matched based on age, sex, and without habit with similar socioeconomic backgrounds	- Biopsy-proven squamous cell cancer of the oral cavity with clinical stage III/IV, with a habit of smoking or tobacco chewing - Healthy adults	N/A	Not given
Gokul et al/2010 ^13^	India	To evaluate the oxidant antioxidant status in blood samples and tumor tissue in OSCC patients in comparison with the healthy controls.	2	1:1	- New cases of histologically proven OSCC (well to moderately differentiated) patients with clinical stage III ⁄ IV, and they were either tobacco chewers or smokers. - Healthy adults without habits.	N/A	Not given
Siddhartha et al/2011 ^14^	India	To examine the serum level of the Sialic acid, total antioxidant, and lipid peroxidation in the head and neck cancer patients.	2	1:1 Age and sex matched. Same socioeconomic status and dietary habits	- Head and neck cancer patients with various clinical changes of oral cancer. - Healthy adults.	N/A	Not given
Marakala et al/2012 ^15^	India	To determine levels of lipid peroxidation and antioxidant vitamin status in patients with oral cavity and oropharyngeal cancer	2	1:1 Matched on basis of age and sex	- Age 40-68 years. Clinically and histopathologically proved oral cavity and oropharyngeal cancer -Normal healthy volunteers.	N/A	Not given
Shetty et al/2014 ^16^	India	To evaluate the salivary MDA levels in subjects with OSCC and PMD.	3 groups HC1-without quid chewing and/or smoking habits, HC2 -with quid chewing and/or smoking habits. PMD-diagnosed cases	2:1	- 20-60 years - Patients diagnosed with OSMF, OL and OSCC. - Healthy patients with a history of habit	N/A	Not given
Srivastava et al/2016 ^17^	KSA	To evaluate lipid peroxidation and antioxidant status in the venous blood of patients with different clinicopathologic stages of leukoplakia.	3	1:1 Matched on the basis of age and sex	New cases of histopathologically proven leukoplakia of various clinical stages.	N/A	Not given
Meera et al/2019 ^18^	India	-To estimate and compare the level of 8-isoprostane in plasma and saliva in patients with OSCC, OSMF, and in controls. -To find out if 8-isoprostane can be used as an effective oxidative stress marker in evaluating the disease progression in OSCC.	3	1:1	-Clinically diagnosed OSMF patients, clinically and histopathologically diagnosed cases of OSCC -Normal patients without any associated habits of smoking, alcohol, or chewing -Age group 15–65 years.	N/A	Not given
Khan et al/2021 ^19^	India	To determine and compare serum and salivary MDA levels to assess the degree of oxidative damage caused by oral leukoplakia and OSCC patients.	3 -1 Control group. - 2 Case groups.	1:1	Newly diagnosed cases of OSCC -Normal healthy individuals -No comorbid.	N/A	1 Year (2016-2017)
Shamsi et al/2023 ^20^	India	to assess the levels of MDA and SOD in the serum of patients diagnosed with OSCC and OSMF.	3 -1 Control group -2 Cases group	1:1	-Age 18 years and above. -Diagnosed cases of OSCC and OSMF - Healthy Controls.	N/A	Not given

N/A: not applicable; OSCC: oral squamous cell carcinoma; OSMF: oral submucous fibrosis; PMD: potentially malignant disorders; OL: oral leukoplakia; MDA: malondialdehyde.

 Almost all the studies were from India, except for one from the Kingdom of Saudi Arabia. All of the studies were case‒control studies, following the cases:control criteria of 1:1, except for one study by Shetty et al. in 2014, which had a 1:2 ratio.^12-20^ Cases were matched with controls based on age, sex, and socioeconomic status,^12-20^ and four studies further matched them based on habits like smoking or chewing tobacco (Table 1).^13,14,16,18,19^

 The oxidative stress markers primarily used were MDA and 8-isoprostane, both of which form as a result of lipid peroxidation. The medium of analysis was primarily serum^12-15,18,20^ and saliva,^16,18,19^ while only one study used only saliva.^16^ Other compounds that have been researched in association with oxidative damage are sialic acid and nitric oxide levels. These markers were used as indicators of malignant transformation, with some supporting the presence of the primary marker.

 Case groups included patients with histologically proven OSCC, OSMF, or leukoplakia, with one study further stratifying the cases by the stage of the pathology into more detailed stages, such as OSCC^14,18^ and leukoplakia.^16^ The controls were healthy participants with no history of habit or comorbid conditions.^12-20^ None of the studies incorporated any intervention of any medications or breaking the habit. None of the studies have defined the data collection time, except for the study by Khan et al,^19^ which was continued for one year. The sample size, diseases studied, and measurement method varied in all the studies (Table 2).^12-20^

**Table 2:** Summarization of parameters of published articles to compare the outcomes of different studies

**Study Design**	**Year/Country**	**Test**	**Medium**	**Biomarker**	**Sample**	**Result** **(mean value)**	***P*** ** value**
Case-control ^12^	2003/India	Colorimetric methods.	Plasma/Serum	LHP and MDA	Controls = 15 Cases = 15	**Controls:** 1. LPH = 290.57 ± 48.70 µmol/mL 2. MDA = 496.29 ± 19.51* nmol/mL) **OSCC:** 1. LPH = 0.0000 2.02 ± 0.23 µmol/mL 2. MDA = 5.57 ± 0.97* nmol/mL)	*P ≤ 0.000 Significant
Case-control ^13^	2010/India	Spectrophotometer	Serum/Salivary	MDA	Controls = 25 Cases (OSCC) = 18	**Serum:** **Controls:** 139.44 ± 22.32 (nmolg^-1^ Hb) OSCC = 159.83 ± 36.39 (nmolg^-1^ Hb) **Saliva:** Controls = 0.68 ± 0.33 nmolmg^-1^ protein) OSCC = 1.12 ± 0.76 (nmolmg^-1^ protein)	*P* = 0.04 Significant
Case-control ^14^	2011/India	TBA Method	Serum	MDA	Control = 50 Cases (OSCC) = 50	C = 3.54 ± 0.09 (µM/L) OSCC = 5.44 ± 0.74 (µM/L)	*P* ≤ 0.0001 Significant
Case-control ^15^	2012/India	Ohkawa et al. method	Serum	MDA	Control = 40 Cases (OSCC & Oropharyngeal C) = 40	C = 486 *(nM/dL) OSCC/OPC = 1827 (nM/dL)	*P ≤ 0.0001 Significant
Case-control ^16^	2014/India	TBA-TCA method	Saliva	Malondialdehyde (MDA)	Control = 65 Cases (Premalignant Disorders) = 115 Cases (OSCC) = 50	C = 0.1812 (nmol/mL) PMD = 0.4259 (nmol/mL) OSCC = 0.9306 (nmol/mL)	*P* = 0.001 Significant
Case-control ^17^	2016/KSA	Ohkawa et al. method	Plasma	TBARS	Control without tobacco chewing (Group III) = 20 Control with tobacco chewing (Group II) = 20 Cases (Leukoplakia) (Group I) = 20	Group III = 1.30 ± 0.40 (nM/mL) Group II = 2.050 ± 0.94* (nM/mL) Group I = 3.30 ± 1.08 (nM/mL)	*P* = 0.001 Significant
Case-control ^18^	2019/India	ELISA	Saliva and Plasma	8-Isoprostane	Control = 10 Cases (OSMF) = 10 Cases (OSCC) = 10	C = 296.5(ng/mL) OSMF = 332.7(ng/mL) OSCC = 492.9(ng/mL)	*P*= 0.037 Significant
Case-control ^19^	2021/India	TBA Method	Serum/Salivary	MDA	Control = 15 Cases (Leukoplakia) = 15 Cases (OSCC) = 15	Saliva:Control = 18.75 (nmol/dL) Leukoplakia = 22.45 (nmol/dL) OSCC = 35.26 (nmol/dL) **Serum:** Control = 3.21 (nmol/mL) Leukoplakia = 4.27 (nmol/mL) OSCC = 6.92 (nmol/mL)	Controls: < 0.005 Leukoplakia: < 0.001 OSCC: 0.001
Case-control ^20^	2023/India	ELISA	Serum	MDA	Control = 20 Cases (OSMF) = 20 Cases (OSCC) = 20	Control = 248.7 (pg/mL) Cases (OSMF) = 403 (pg/mL) Cases (OSCC) = 469.3 (pg/mL)	*P*≤ 0.0001 Significant

LHP: lipid hydroperoxide; MDA: malondialdehyde; OSCC: oral squamous cell carcinoma; OSMF: oral submucous fibrosis.

 Since the diseases studied were varied, i.e., OSCC and/or leukoplakia and OSMF, the results were compared according to the similarity of cases. Meera et al^18^ and Shetty et al^16^ reported increased oxidative stress markers, including 8-isoprostane and MDA levels, in OSCC and OSMF compared to controls, indicating that these markers were associated with the disease progression. Srivastava et al^17^ and Siddhartha et al^14^ observed significant differences in lipid peroxidation and antioxidant status between patients with leukoplakia and those with head and neck cancer, establishing the involvement of oxidative stress in the pathogenesis of these diseases. Beevi et al^12^ and Gokul et al^13^ found a correlation between higher clinical stages of OSCC and augmented levels of oxidative stress. Khan et al^19^ and Shamsi et al^20^ also established higher concentrations of MDA in the serum and saliva of patients with OSCC and leukoplakia compared to the controls.Marakala et al^15^ reported alterations in lipid peroxidation and vitamin antioxidants of patients with oral and oropharyngeal cancer.

## Discussion

 The main aim of this systematic review was to elucidate the role of oxidative stress biomarkers in the progression of lesions to premalignant and then to malignant conditions and to provide a better understanding of how to effectively use them for early diagnosis/recurrence in clinical settings. This will improve the lives and benefit the patients in low-resource countries with a high number of betel nut and tobacco consumers. We analyzed data from the available literature fulfilling the criteria set by the investigators and tabulated the data from nine studies conducted in India and Saudi Arabia. We observed consistent evidence supporting the use of oxidative stress markers and antioxidant status as diagnostic and prognostic tools.

**Figure 2:**
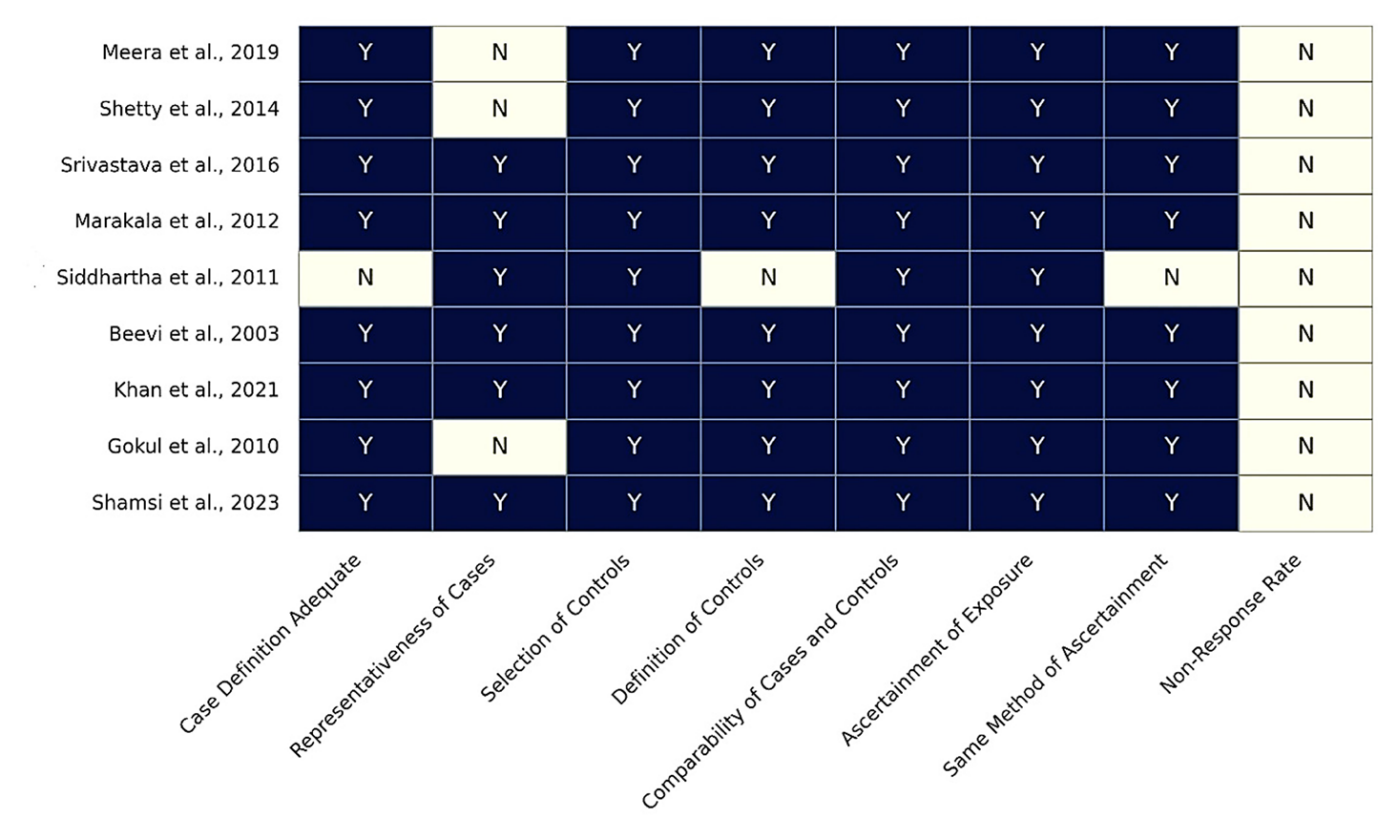
Risk of bias assessment of studies using the Newcastle-Ottawa Scale (NOS)

 During the evaluation of studies included in the systematic review, the risk of bias was calculated for each study using the NOS tool for case‒control studies. It was found that none of the studies mentioned the “non-response rate” (Figure 2), possibly because these diagnostic studies did not require patient follow-up, thus omitting non-response rates. Furthermore, none of the studies mentioned the study duration in the articles. They mainly included the diagnosed patients in cases. Three studies underrepresented the cases, showing a 1:1 case-to-control ratio, but failed to follow up.^17-19^ In addition, the study by Siddhartha et al^14^ demonstrated several shortcomings across multiple NOS domains. These methodological flaws weaken the overall validity of the evidence, suggesting that the apparent consistency of results across studies should be interpreted with caution. Except for these issues, the remaining eight studies generally showed low risk of bias according to the NOS tool.

 The majority of the studies only considered age and sex^13,15-20^ when selecting controls, while only a few extended this to include socioeconomic status of the patients as well.^12,14^ The matching of cases and controls based on these factors helped minimize confounding factors and strengthen the reliability of the findings. This review underscores the role of oxidative stress markers in the progression of oral lesions from premalignant to malignant stages. Their clinical application could enhance early diagnosis and recurrence monitoring, particularly in resource-limited regions with high consumption of betel nuts. However, most studies were conducted in India, which limits their generalizability. Further large-scale studies are needed for validation.^12-15,17,19,20^

 Several studies highlighted the impact of habits, including smoking, alcohol consumption, and betel quid chewing, on oxidative stress levels. Shetty et al^16^ differentiated between habitual and non-habitual groups, revealing that individuals with tobacco-related habits exhibited higher oxidative stress even in the absence of clinical disease. These findings highlight that the rise of markers may not be limited to changes in carcinoma, and early intervention in such cases will be redundant. However, this can help identify high-risk populations for targeted preventive measures.

 Oxidative stress plays a significant role in neurodegenerative diseases and conversion from premalignant to malignant pathologies. It is basically the imbalance between reactive oxygen species (ROS) production as a result of injury or trauma caused by exposure to carcinogens and the antioxidant defenses, which decrease free radicals and play a critical role in the initiation and progression of oral dysplasia, ultimately leading to carcinogenesis. Lipid peroxidation products formed due to oxidative stress, such as MDA^12-16,19,20^ and 8-isoprostane,^18^ initially showed promise as biomarkers in the reviewed studies. Elevated levels of these markers were compared in healthy participants and patients with premalignant lesions and OSCC. This was demonstrated by Meera et al^18^ and Shetty et al,^16^ which underscores the increased oxidative damage in OSCC and OSMF patients compared to healthy controls.

 The findings from the studied literature align with established theories, which support the notion that ROS can induce DNA damage, protein oxidation, and lipid peroxidation, resulting in genetic instability, thus promoting carcinogenesis. Another study by Srivastava et al^17^ further enhanced the findings by observing elevated oxidative stress in precancerous conditions such as leukoplakia, which consists of several types of lesions. This also suggests its role as an early event in the malignant transformation process.

 The reviewed articles also evaluated the antioxidant defense systems that prevent oxidative damage. Decreased levels of antioxidants in OSCC and other premalignant lesions, as reported by Siddhartha et al^14^ and Gokul et al,^13^ point towards a compromised defense mechanism in these patients. This reduction in antioxidant capacity, coupled with elevated oxidative stress markers, emphasizes the oxidative‒antioxidative imbalance in these diseases. The depletion of antioxidants could be attributed to chronic ROS exposure in the presence of predisposing factors such as tobacco chewing, smoking, and alcohol consumption. Beevi et al^12^ demonstrated that patients with advanced clinical stages of OSCC exhibited significantly higher oxidative stress levels compared to controls, highlighting the association between oxidative stress and disease severity. Similarly, Gokul et al^13^ confirmed this correlation by showing elevated oxidant–antioxidant imbalance in both blood and tumor tissue of OSCC patients. In a separate study, Khan et al^19^ reported that patients with OSCC and leukoplakia had significantly higher serum and salivary MDA concentrations compared to healthy individuals. Shamsi et al^20^ further validated these findings by documenting increased serum levels of MDA and SOD in patients with OSCC and OSMF. Additionally, Marakala et al^15^ highlighted alterations in lipid peroxidation and antioxidant vitamin status in patients with oral and/or oropharyngeal cancers, underscoring the systemic oxidative stress burden in these populations.

 An overall review of the literature studied collectively suggests that oxidative stress markers, particularly MDA and 8-isoprostane, have diagnostic and prognostic relevance in managing oral diseases, particularly premalignant and malignant pathologies. Elevated MDA levels, as reported by Khan et al^19^ and Shamsi et al,^20^ correlate with the severity of OSCC and leukoplakia.These findings reinforce the potential of MDA as a reliable marker for assessing disease burden and monitoring progression. Similarly, 8-isoprostane, evaluated by Meera et al,^18^ demonstrates promise as an oxidative stress marker, particularly for OSCC and OSMF.However, its use in clinical practice requires further validation through large-scale studies.

 The other problem with these markers is that the extraction medium, saliva, has its own set of limitations. In cases of already treated oral cancer patients, there is a chance of salivary flow reduction due to radiotherapy, medications, or systemic conditions.^21^ However, they are cheap, non-invasive, and safe in comparison to serum. Thus, it is concluded that lipid peroxidation products not only indicate oxidative damage but also signify the cumulative effect of environmental and lifestyle risk factors like smoking, which are prevalent in oral cancer etiology.

## Conclusion

 This systematic review consolidates the evidence supporting the role of oxidative stress biomarkers in the pathogenesis and progression of oral diseases. Although oxidative stress markers such as MDA and 8-isoprostane consistently showed diagnostic potential, the predominance of studies from India limits the generalizability of these findings. Further large-scale, multicenter research across diverse populations is essential to establish the true clinical applicability of these findings. Addressing oxidative stress through targeted therapies may improve patient outcomes.

## Competing Interests

 The authors declare that they have no conflicts of interest.

## Ethical Approval

 This study was registered with PROSPERO (CRD42024565417).
